# Evaluation of sleep apnea syndrome risk in patients attending a smoking cessation outpatient clinic

**DOI:** 10.18332/tid/218371

**Published:** 2026-03-23

**Authors:** Meryem Betos Koçak, Derya Aydin

**Affiliations:** 1 Department of Family Medicine, Health Sciences University, Faculty of Medicine, Balıkesir Atatürk City Hospital, Balıkesir, Turkey; 2 Department of Chest Diseases, Balıkesir Atatürk City Hospital, Balıkesir, Turkey

**Keywords:** smoking cessation, sleep apnea syndrome, cigarette addiction

## Abstract

**INTRODUCTION:**

Smoking is a major public health issue with undeniable adverse effects on human health. It is associated with a wide range of diseases, including cancer, cardiovascular conditions, pulmonary disorders, and neurological diseases. Among these, sleep disturbances and respiratory tract disorders, particularly Obstructive Sleep Apnea Syndrome (OSAS), are of particular concern. This study aims to investigate the relationship between smoking cessation and the OSAS risk scores.

**METHODS:**

Our study is a prospective, pre-post, single-arm observational study with repeated measures, conducted on patients who successfully quit smoking (n=117). The Epworth Sleepiness Scale, Berlin Questionnaire, and STOP-BANG Questionnaire were utilized to assess the risk of OSAS. OSAS risk scores were obtained from patients at their initial visit to the smoking cessation clinic and again six months after smoking cessation.

**RESULTS:**

Our study was completed with 117 patients. According to the Epworth Sleepiness Scale, high daytime sleepiness was reported in 36 patients, while quitting smoking reduced this number to 30 patients. According to the Berlin Questionnaire, the number of high-risk patients, which was 47, decreased to 28 after smoking cessation. According to the STOP-BANG Questionnaire, the rate of patients considered to be at high risk for OSAS was 45.3%, but it decreased to 35.9% after smoking cessation.

**CONCLUSIONS:**

Our study demonstrated that the number of high-risk patients, as determined by OSAS risk scales, decreased after smoking cessation. Our study provides indications that the risk of OSAS may decrease with smoking cessation.

## INTRODUCTION

Tobacco use represents a major global public health concern, being associated with over 8 million deaths annually worldwide. It is also estimated to impose an economic burden of approximately US$1.4 trillion on the global economy each year. Smoking is linked to a wide range of diseases such as respiratory illnesses, coronary heart disease and cancer^1^. It continues to be one of the main causes of preventable sickness and death around the world. The earlier an individual quits smoking, the lower their risk of developing smoking-related diseases. Even cessation at an advanced age results in substantial health improvements. Despite the availability of various treatments to support smoking cessation, success rates remain insufficient^2^. Given that the adverse health effects of smoking are, to a considerable extent, reversible, smoking cessation has become a prominent area of research and is actively supported by various governmental bodies^3^.

Many individuals who use tobacco report a desire to cease smoking; however, nicotine dependence presents a significant barrier to successful cessation^4^. As a result, various treatments, such as nicotine replacement therapies, nicotine receptor agonists, and antidepressant medications, are employed to aid smoking cessation^1^. Nicotine increases the release of dopamine in the brain, contributing to addiction^5^. Cigarettes contain approximately 7000 chemicals, 60 to 70 of which are known human carcinogens. While nicotine is the primary agent responsible for cigarette addiction, the by-products of smoking are responsible for tobacco-related diseases and mortality^3^.

Quitting smoking before the age of 40 years reduces the risk of death from smoking-related diseases by approximately 90%^6^. Chronic diseases related to smoking typically develop after several decades of smoking. Smokers face a 25-fold increased risk of lung cancer compared to non-smokers, and the risk of coronary heart disease or stroke is 2 to 4 times higher in smokers than in non-smokers^7^.

Smoking is responsible for a wide range of diseases, including Obstructive Sleep Apnea Syndrome (OSAS)^8^ an association that has been demonstrated in numerous studies^9^. The present study aimed to evaluate the potential impact of smoking cessation on the probability of developing OSAS as assessed through risk scores.

## METHODS

### Study design and participants

This was a prospective, pre-post, single-arm observational study with repeated measures. Our study was conducted on individuals seeking treatment at the Smoking Cessation Outpatient Clinic of Balikesir Atatürk City Hospital, in Turkey. The study included patients who visited the clinic between 1 January and 31 December 2024, and successfully quit smoking after receiving treatment.

The inclusion criteria comprise individuals aged 18–65 years who present at a smoking cessation outpatient clinic and successfully quit smoking with the treatment provided. Exclusion criteria include patients for whom medication is considered inappropriate by the physician, those who refuse medication, and individuals who do not adhere to or discontinue the prescribed smoking cessation treatment. Additional exclusions are patients who fail to attend follow-up appointments, have incomplete or incorrect medical records, are pregnant or breastfeeding, or have diagnosed sleep disorders. Patients with neuromuscular diseases, alcohol use, or known pulmonary conditions such as asthma, COPD, bronchiectasis, or OSAS are also excluded.

Sample size calculations were performed using G*Power 3.1.9.7 software. A medium effect size (Cohen’s d=0.5), a 5% significance level (α=0.05), and a 90% test power (1–β = 0.90) were determined as criteria for the analysis. Based on these parameters, a sample size of at least 44 participants was determined to be sufficient to detect a statistically significant difference.

Participation in the study was voluntary, and all patients gave their informed consent. Our study was approved by the local ethics committee Balıkesir Atatürk City Hospital Ethics Committee, Date: 21.12.2023, Decision No: 2023/12/78 and conducted in accordance with the principles of the Helsinki Declaration.

**Figure 1:**
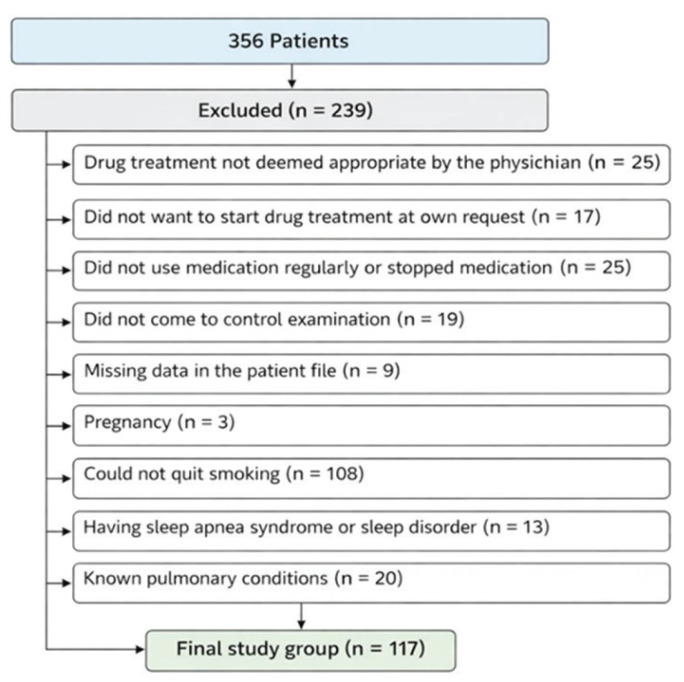
Flowchart of participants selection and exclusion

Our study was conducted on patients who presented to the smoking cessation outpatient clinic and successfully quit smoking. During the study period, a total of 356 patients presented to the clinic, but after applying the exclusion criteria, the study was completed with 117 patients. The flowchart of the study is shown in Figure 1.

### Procedure

Study data were obtained from standardized forms completed individually for each patient. These forms were filled in by the attending physician for every individual who presented to the smoking cessation outpatient clinic. The clinic is staffed by physicians authorized by the Republic of Türkiye Ministry of Health, and all patients are treated by certified physicians.

During the study, all patients received bupropion as part of their medical treatment, following the same procedure. The treatment was administered at a standard dosage of 150 mg/day for the first 3 days and 300 mg/day for the 4th to 30th day for one month. The smoking cessation status of all patients at the end of treatment was recorded. Patients who successfully quit smoking were invited for a follow-up appointment six months after cessation, at which point the same scales were completed again. The data obtained at the follow-up at six months were recorded on the study form for each patient. Smoking cessation was verified using exhaled carbon monoxide measurement (CO <4 ppm).

### Data collection tools

A study form was developed for data collection, which was completed by a physician. The demographic data of the participants included in the study, such as age, gender, and comorbidities (cardiovascular diseases such as ischemic heart disease, heart failure, and arrhythmia; pulmonary diseases such as COPD, pulmonary fibrosis, bronchiectasis, and atelectasis), hypertension, and diabetes, were recorded. Smoking habits were classified based on daily cigarette consumption: 0–10, 11–20, 21–30, and >30 . The duration of smoking was categorized as 0–10, 11–20, 21–30, and >30 years. Additionally, vital signs such as blood pressure, pulse rate, temperature, respiratory rate, and oxygen saturation were recorded. Passive exposure to cigarette smoke was also evaluated, and individuals with documented secondhand smoke exposure were excluded from the study, as such exposure was considered a potential confounding factor in assessing complete smoking cessation. The scale values were obtained through face-to-face interviews conducted by a physician.

The Epworth Sleepiness Scale was used to assess daytime sleepiness. The scale consists of 8 questions, to which individuals are asked to assign a score ranging from 0 to 3. A score of 0 indicates ‘I never doze off’, 1 indicates ‘I sometimes doze off’, 2 indicates ‘I usually doze off’, and 3 indicates ‘I definitely doze off’. A score ≤10 is classified as normal sleepiness, a score 10–16 is considered increased sleepiness, and a score ≥16 is classified as pathological sleepiness. A Turkish validity and reliability study of the Epworth Sleepiness Scale has been conducted^10^. Patients who scored ≥10 on the Epworth Sleepiness Scale were considered to be at high probability for daytime sleepiness.

The Berlin Questionnaire was used as a tool for community screening of OSAS. It is composed of 3 categories and includes a total of 10 questions: 5 questions in Category 1; 4 questions in Category 2; and 1 question in Category 3. A score ≥2 in Categories 1 and 2 is considered positive for that category, while a score of 1 in Category 3 is considered positive. If at least two of the three categories are positive, the individual is classified as high probability; if one or none of the categories is positive, the individual is classified as low probability. A Turkish validity and reliability study of the Berlin Questionnaire has been conducted^11^. Patients who test positive in two or more categories of the Berlin Questionnaire were considered to be at high probability for Sleep Apnea Syndrome.

The STOP-BANG Questionnaire was also used to assess the presence of Obstructive Sleep Apnea Syndrome (OSAS). It is derived by adding four additional questions to the original STOP questionnaire. Each ‘yes’ answer to the questions is assigned a score of 1 point. A total score ≥3 points is considered indicative of high probability. A Turkish reliability and validity study of the STOP-BANG questionnaire is available^12^. Patients who scored ≥3 on the STOP-BANG scale were classified as high-probability for Sleep Apnea Syndrome.

The Fagerström test for nicotine dependence was also administered at the time of presentation to the smoking cessation outpatient clinic. In addition, values from the Epworth Sleepiness Scale, Berlin Questionnaire, and STOP-BANG Questionnaire were obtained at the time of admission and again six months after smoking cessation. All data were prospectively recorded on individual study forms created for each patient during their initial visit to the smoking cessation outpatient clinic.

### Statistical analysis

All statistical analyses were performed using IBM SPSS Statistics version 23.0 (IBM Corp., Armonk, NY, USA). Descriptive data are presented as mean ± standard deviation (SD), median (minimum–maximum), and frequency (n) with percentages (%), as appropriate. The normality of continuous variables was assessed using the Shapiro–Wilk and Kolmogorov–Smirnov tests. Categorical variables were compared using the chi-squared test or Fisher’s exact test, as appropriate. For within-group comparisons of pre- and post-procedure measurements, the paired samples t-test was used for normally distributed data, and the Wilcoxon signed-rank test was applied for non-normally distributed data. A two-tailed p<0.05 was considered statistically significant for all analyses.

## RESULTS

All patients tolerated bupropion treatment. The patients included in the study were aged 19–65 years, with a mean age of 43.8 (SD=12.6) years. Of the participants, 76 (65%) were male. The comorbidities of the patients were as follows: 78 patients (66.7%) had cardiovascular disease, 35 patients (29.9%) had pulmonary disease, 20 patients (17.1%) had hypertension, and 15 patients (12.8%) had diabetes mellitus.

The daily cigarette consumption of the participants was as follows: 5 (4.3%) smoked 1–10; 38 (32.5%) smoked 11–20; 58 (49.6%) smoked 21–30; and 16 (13.7%) smoked >30. The duration of smoking was distributed as follows: 36 patients (30.8%) had smoked for 0–10 years, 31 patients (26.5%) had smoked for 11–20 years, 35 patients (29.9%) had smoked for 21–30 years, and 15 patients (12.8%) had smoked for >30 years. According to the Fagerström nicotine dependence scale, 1 (0.9%) patient was classified as very low dependence, 7 (6%) patients were classified as low dependence, 3 (2.6%) patients were classified as moderately dependent, 34 (29.1%) patients were classified as highly dependent, and 72 (61.5%) patients were classified as very highly dependent.

At the time of recruitment, 36 (30.8%) patients were considered at a high probability for daytime sleepiness according to the Epworth Sleepiness Scale, while six months after smoking cessation, the number of patients considered to be of high probability for daytime sleepiness decreased to 30 (25.6%) (p<0.05). Regarding the Berlin Questionnaire, used for OSAS screening, 47 (40.2%) patients were classified as high probability at the time of recruitment while this number decreased to 28 (23.9%) six months after smoking cessation, which was statistically significant (p<0.05). Similarly, on the STOP-BANG Questionnaire, 53 (45.3%) patients were classified as of high probability at the time of presentation while on the other hand, after six months of smoking cessation, the number of high probability patients decreased to 42 (35.9%), which was statistically significant (p<0.05) as shown in Table 1.

**Table 1:** Comparison of scales used in the study before and after quitting smoking (N=117)

*Scales*	*While smoking high risk* *n (%)*	*After quitting smoking high risk* *n (%)*	p*
Epworth Sleepiness Scale	36 (30.8)	30 (25.6)	<0.05
Berlin Questionnaire	47 (40.2)	28 (23.9)	<0.05
STOP-BANG Questionnaire	53 (45.3)	42 (35.9)	<0.05

* Paired samples t-test.

## DISCUSSION

Our study was conducted on patients who successfully quit smoking. Initially, the scales assessing the probability of OSAS were administered to all patients at the time of presentation to the smoking cessation outpatient clinic, while they were still actively smoking. Afterward, patients who had quit smoking were invited for a follow-up visit 6 months later, at which time the OSAS probability scales were readministered, confirming they had not smoked for the entire 6-month period. Our findings indicate that the probability of OSAS was lower after smoking cessation compared to the period of active smoking.

OSAS is more common in men than in women, with a prevalence of 24% in men and 9% in women^13^. OSAS is defined by repeated episodes of respiratory arrest, reduced blood oxygen levels, and brief awakenings caused by partial or complete blockage of the upper airways during sleep. Risk factors for OSAS involve anatomical abnormalities that narrow the upper airway, older age, obesity, being male, use of drugs or substances that decrease muscle tone, and hormonal disorders^13^. The Wisconsin Sleep Cohort study reported that smokers experienced greater difficulty initiating and maintaining sleep compared to non-smokers. It also demonstrated that smokers had more trouble waking up in the morning and were more prone to poor sleep quality, including symptoms such as non-restful sleep^8^. Several theories have been proposed to explain the relationship between smoking and Obstructive Sleep Apnea Syndrome (OSAS). These theories include: changes in sleep architecture, muscle relaxation and nerve reflex alterations due to nicotine, an increased arousal threshold during sleep due to nicotine, and upper respiratory tract inflammation caused by smoking^14^.

Changes in sleep architecture are characterized by prolonged sleep onset, frequent awakenings, and poor sleep quality^14^. A study utilizing polysomnography found that smokers had increased rapid eye movement sleep but experienced shorter total sleep duration and longer sleep latency^15^. These disruptions to the sleep cycle contribute to the development of OSAS^14^.

Chronic exposure to smoking leads to inflammation in the upper airway mucosa through the action of calcitonin gene-related peptide (CGRP). When this inflammation becomes chronic, it results in impaired ciliary function, mucosal edema, cellular proliferation, and thickened epithelium, all of which are considered contributing factors to the onset of OSAS^16,17^.

Yosunkaya et al.^13^ demonstrated that the majority of patients with severe OSAS were smokers, and smoking contributed to an increase in the severity of OSAS by elevating the frequency of apneas and hypopneas. Smoking also worsens nocturnal oxygenation by affecting the lower respiratory tract^13^. A systematic review and meta-analysis further confirmed the strong association between smoking and OSAS, and noted that heavy smokers were at a higher probability for OSAS and patients with severe OSAS smoked more than those with mild and moderate OSAS^8^. While the mechanisms by which smoking may contribute to OSAS have been investigated, there is limited information on the impact of smoking cessation on reversing these mechanisms^14^.

Lung functional capacity improves with smoking cessation. In a study by Pezzuto et al.^18^ oxygen saturation (SaO2), oxygen pressure (PaO2), and forced expiratory volume in 1 second increased after smoking cessation, while the oxygen desaturation index decreased and the distance covered in the walking test increased. Generally, it is predicted that OSAS symptoms will improve following smoking cessation, given the finding that the sleep quality of former smokers is better than that of active smokers^19^. Zeng et al.^8^ highlighted the lack of data linking OSAS outcomes to smoking cessation and emphasized the need for prospective studies on this topic. Krishnan et al.^14^ also noted the necessity of studies evaluating OSAS in patients who quit smoking.

### Limitations

Our study has some limitations. Some of the limitations include its non-randomized design, non-causal design, and limited generalizability to other countries. Other limitations include the absence of a control group, and the potential for the contributing role of other factors which may mediate or confound this relationship such as age, BMI, and comorbidities.

## CONCLUSIONS

Smoking is linked to numerous life-threatening diseases, yet many conditions can be reversed following smoking cessation. In our observational study, we investigated the relationship between OSAS and smoking. The data presented in our article suggest that the probability of OSAS decreases after smoking cessation.

## Data Availability

The data supporting this research are available from the authors on reasonable request.
